# Differential Effects of Unihemispheric Concurrent Dual-Site and Conventional tDCS on Motor Learning: A Randomized, Sham-Controlled Study

**DOI:** 10.32598/bcn.9.10.350

**Published:** 2019-01-01

**Authors:** Ailin Talimkhani, Iraj Abdollahi, Mohammad Ali Mohseni-Bandpei, Fatemeh Ehsani, Sanaz Khalili, Shapour Jaberzadeh

**Affiliations:** 1. Department of Physical Therapy, School of Rehabilitation Sciences, Hamadan University of Medical Sciences, Hamadan, Iran.; 2. Department of Physical Therapy, University of Social Welfare and Rehabilitation Sciences, Tehran, Iran.; 3. Pediatric Neurorehabilitation Research Center, University of Social Welfare and Rehabilitation Sciences, Tehran, Iran.; 4. University Institute of Physical Therapy, Faculty of Allied Health Sciences, University of Lahore, Lahore, Pakistan.; 5. Neuromuscular Rehabilitation Research Center, Semnan University of Medical Sciences, Semnan, Iran.; 6. Department of Biostatistics and Epidemiology, School of Public Health, Hamadan University of Medical Sciences, Hamadan, Iran.; 7. Non-invasive Brain Stimulation & Neuroplasticity Laboratory, Monash University, Melbourne, Australia.; 8. Department of Physiotherapy, School of Primary and Allied Health Care, Faculty of Medicine, Nursing and Health Sciences, Monash University, Melbourne, Australia.

**Keywords:** Transcranial direct current stimulation, Primary motor cortex, Dorsolateral prefrontal cortex, Learning, Motor skills

## Abstract

**Introduction:**

Based on the literature, unihemispheric concurrent dual-site anodal transcranial Direct Current Stimulation (a-tDCSUHCDS) of primary Motor cortex (M1) and Dorsolateral Prefrontal Cortex (DLPFC) would be more efficient than conventional a-tDCS of M1 to induce larger and longer-lasting M1 corticospinal excitability. The main objective of the present study was to compare the effects of a-tDCSUHCDS and conventional M1 a-tDCS on the extent and durability of the motor sequence acquisition in healthy individuals.

**Methods:**

In this randomized sham-controlled study, healthy volunteers were randomly divided into three groups: experimental (a-tDCSUHCDS), control (M1 a-tDCS), and sham stimulation groups. The participants practiced serial response time task over three consecutive days when they simultaneously received a-tDCS. Using the skill measure, we assessed motor learning up to 4 weeks after the completion of experimental conditions.

**Results:**

Data analysis revealed that all groups exhibited the improved trend over the training course (P<0.001). There were no significant differences in skill acquisition among groups at post-intervention (P>0.05), while a significant improvement was observed between experimental and sham group at the retention time (P<0.05). Moreover, there were no significant differences between the control and two other groups with regard to the retention time (P>0.05).

**Conclusion:**

These results revealed a significant increase in the skill acquisition by a-tDCSUHCDS technique with regard to retention issue, which could be a valuable finding in neuro-rehabilitation field.

## Highlights

- Motor skill learning is remarkably improved immediately after multiple sessions of simultaneous transcranial direct current stimulation and training in healthy individuals.
- In our study, the effects of multiple sessions of unihemispheric concurrent dual-site anodal transcranial direct current stimulation during training lasted up to 4 weeks.
- Unihemispheric concurrent dual-site anodal transcranial direct current stimulation improves motor learning more profoundly than conventional and sham transcranial direct current stimulation over the follow-up period of 4 weeks.

## Plain Language Summary

Nowadays, transcranial direct current stimulation (tDCS) is a useful complementary therapy to enhance motor skill learning in the stroke patients. However, unihemispheric concurrent dual-site a-tDCS (a-tDCSUHCDS) as a new technique induces long-lasting and more positive outcomes as compared to conventional primary motor cortex (M1) tDCS. In this study, multiple sessions of a-tDCSUHCDS, conventional a-tDCSM1 and sham tDCS groups were studied and the acquired motor skill learning were compared among the groups up to 4 weeks after the completion of the stimulation. The findings indicate that a-tDCSUHCDS profoundly enhanced the size and lasting duration of the motor skill learning up to 4 weeks, while no significant differences were found between M1 and sham a-tDCS, and between M1 a-tDCS and a-tDCSUHCDS in long-term retention. However, the effect size of a-tDCS was moderate between a-tDCSUHCDS and M1 a-tDCS groups at 4 weeks retention time. Our findings supported more effectiveness of a-tDCSUHCDS technique for induction of prolonged and larger after-effects compared to conventional a-tDCSM1 technique in the young healthy individuals.

## 1. Introduction

Activities of Daily Living (ADL) in individuals is particularly affected by learning motor skills (Dayan & Cohen, 2011), which is considered an essential component in the rehabilitation of patients with neurological disorders, such as stroke patients (Schreiber et al., 2001). Motor learning leads to relatively permanent changes in the individuals’ motor behaviors (Willingham, 1998). One or more sessions of practice as online or offline learning can cause learning gains (Reis et al., 2009; Robertson, Press, & Pascual-Leone, 2005).

Learning gains can alter the functional properties of different brain areas (Karni et al., 1998). According to the results of relevant studies, motor learning and motor cortex plasticity are strongly correlated (Rosenkranz, Kacar, & Rothwell, 2007; Ziemann, Iliać, Pauli, Meintzschel, & Ruge, 2004). The motor learning process promotes synaptic connectivity within the primary Motor cortex (M1) and premotor areas (Dayan & Cohen, 2011; Floyer-Lea & Matthews, 2005). According to the literature, Corticospinal Excitability (CSE) (Nitsche & Paulus, 2000, 2001) and motor learning could be promoted by brain stimulation techniques like transcranial Direct Current Stimulation (tDCS).

Based on some studies, motor learning in many motor skill tasks, such as finger sequencing task (Stagg et al., 2011), Serial Response Time Task (SRTT) (Ehsani, Bakhtiary, Jaberzadeh, Talimkhani, & Hajihasani, 2016; Nitsche et al., 2003), and sequence tapping task (Kantak, Mummidisetty, & Stinear, 2012; Tecchio et al., 2010) can be improved by employing one session of a-tDCS over M1. Moreover, multiple sessions of M1 a-tDCS have been reported to have positive effects on motor learning (Reis et al., 2015; Reis et al., 2009; Saucedo Marquez, Zhang, Swinnen, Meesen, & Wenderoth, 2013; Schambra et al., 2011). Consistent with the relevant literature, the size and duration of the resulted cortical and behavioral changes are influenced by the number of stimulation sessions (Bastani & Jaberzadeh, 2013; Reis et al., 2009; Saucedo Marquez et al., 2013; Vaseghi, Zoghi, & Jaberzadeh, 2015a).

It should be also noted that electrode montage as one of the main tDCS parameters could affect CSE changes and improve motor learning. As reported by Vaseghi et al. (2015a), larger and longer-lasting M1 CSE can be induced by unihemispheric concurrent dual-site a-tDCS (a-tDCSUHCDS) of M1-dorsolateral prefrontal cortex (DLPFC) than conventional a-tDCS of M1. In other words, a-tDCSUHCDS is more efficient in the size and duration of the resulted M1 CSE enhancement (Vaseghi et al., 2015a). Besides, this new approach is superior, because of its high application that can be attributed to the simultaneous stimulation of M1 and DLPFC considering their functional connectivity (Vaseghi et al., 2015a). Therefore, we can hypothesize that multiple sessions of a-tDCSUHCDS could enhance the size and duration of a-tDCS effects on motor learning more than what was achieved by the conventional methods.

The first objective of the present study is to compare the effects of a-tDCSUHCDS and conventional M1 a-tDCS on the extent and durability of the motor sequence acquisition in healthy individuals. The second objective is to investigate on how long the effects would last.

## 2. Methods

### 2.1. Study participants

The present study was carried out on 67 healthy individuals (45 female and 22 male students of University of Social Welfare and Rehabilitation Sciences, Tehran, Iran). Their Mean±SD age was 28.07±3.73 years (range: 19 to 35 years). They were selected from the enrolled students by simple, non-probability sampling method.

The study inclusion criteria included lack of previous musculoskeletal disorders, auditory or visual problems, psychiatric or neurological diseases, or memory or perceptual problems (Mini-Mental State Examination [MMSE] >23 out of 30). The study exclusion criteria included tDCS application contraindications like skin diseases in the areas which could be stimulated, brain tumor, intracranial metal implantation, medications for any neurological disease, or epilepsy.

According to the Edinburgh Handedness Inventory (20-item inventory) (Oldfield, 1971), all individuals were right-handed. All participants were evaluated by a physician before the study. Also, they all signed the written, informed consent forms.

**Figure 1.**
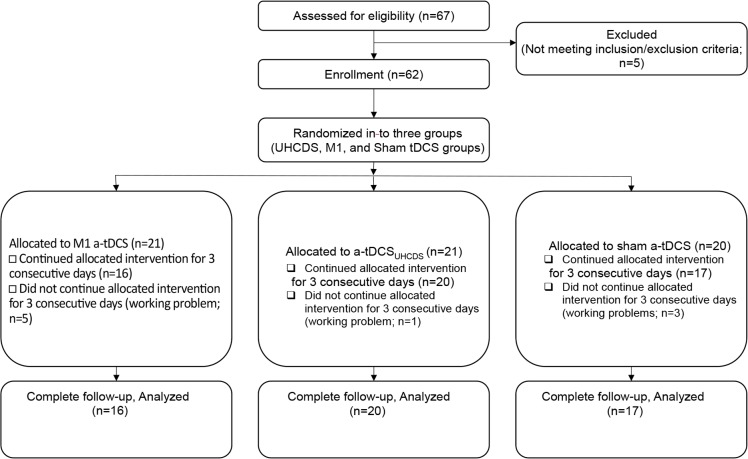
Flow diagram The study participants’ eligibility assessment, enrollment, group allocation and analysis

The CONSORT (Consolidated Standards of Reporting Trials) checklist criteria were met in the present study. Five individuals were excluded from the study because they did not observe the inclusion criteria. A pilot study was carried out on 9 participants, and the results indicated that a sample size of 16 in each group had a power of 80% and Confidence Interval (CI) of 95%. Random number assignments were used to assign 62 enrolled participants into three groups: an experimental group (n=21) that received a-tDCSUHCDS of M1-DLPFC, a control group (n=21) that received atDCS of M1, and a sham group (n=20) that received sham a-tDCS (Figure 1).

As shown in Figure 1, because of their personal reasons, 9 participants failed to complete all three sessions of the study. Finally, 20 experimental, 16 control, and 17 sham participants underwent all sessions of the study, and only the data retrieved from these participants were used in the statistical analysis (Figure 1).

### 2.2. Study design

**Figure 2.**
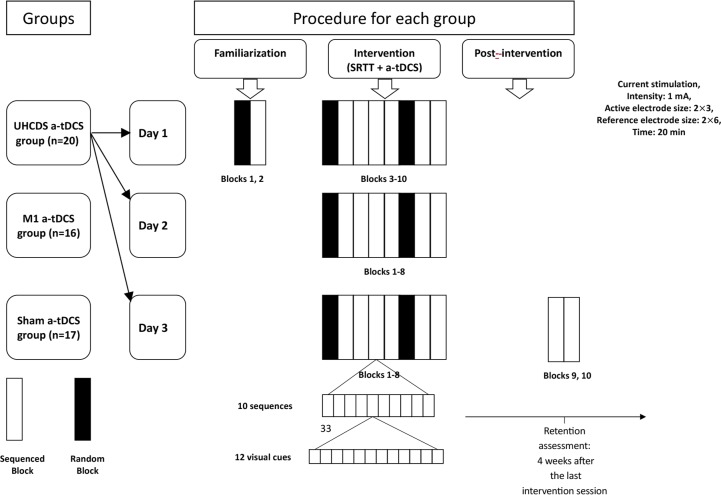
Experimental set-up The participants randomly received a-tDCS (a-tDCSUHCDS, M1, or sham a- tDCS) during three consecutive daily sessions of SRTT and participated in retention assessment, too. Day 1 started with two blocks of random and predetermined sequential order followed by 20 min of stimulation combined with 8 training blocks. Days 2 and 3 of the training only included 8 training blocks during 20 min of stimulation. Both control and sham groups performed the same procedure to the a-tDCSUHCDS group. SRTT consisted of 8 sequential and random blocks. Each block included 10 trials and each trial included 12 stimuli. The retention assessment included two sequence blocks applied for 4 weeks after the last intervention session.

The present study was a randomized, single-blind, sham-controlled study. Each group attended three consecutive daily learning sessions under the aforementioned experimental conditions at the same time each day (Figure 2). The participants were presented with the same instruction. In addition, the participants were blinded to the type of atDCS (active or sham). During the 3 consecutive daily sessions, skill index per each block was calculated.

Four weeks after the last training session, the follow-up assessment was scheduled. This study was confirmed by Human Ethics Committee of the University of Social Welfare and Rehabilitation Sciences, Tehran, Iran, which is compatible with the Declaration of Helsinki. After the enrolment of all participants, the study was recorded as a clinical trial study on the website^1^ of Iranian Registry of Clinical Trials (The registration number: IRCT2016071028808N2).

### 2.3. Serial Response Time Task (SRTT)

Serial Response Time Task (SRTT) is one of the most common tools to assess implicit motor learning (Robertson, 2007). It was programmed using SuperLab 5 (Cedrus, San Pedro, CA, USA). In this task, the unpredicted repeating patterns of visual cues are presented. Each cue is a small black circle (3.5 cm in diameter) that appears at any one of four positions arranged horizontally (left, middle left, middle right, right) on a computer monitor. The participants were taught to press one of the four buttons of response pad (RB-740, Cedrus Corporation, San Pedro, CA, USA) as soon as the circle appeared on the screen using one of the four fingers. They are taught to press button 1 with the index finger when the black circle appears at the left side of the screen, button 2 with the middle finger when the circle appears at middle-left side of the screen, button 3 with the ring finger and button 4 with the small finger, when the circle appears at middle-right or right side of the screen, respectively.

The training task consisted of 8 blocks of visual cues with a rest time of 20 seconds between each block. Of the eight training blocks, two blocks consisted of random sequences of key-press stimuli (block 1 and 6), whereas other blocks (2, 3, 4, 5, 7 and 8) contained repeating sequences. Each block consisted of 10 trials and each trial included 12 stimuli with the following visual cues: 1–4–2–1–3–4–2– 1–3–2–4–3. The target circle would disappear, as soon as the correct key is pressed. Then, after some predefined time (500 ms), the next circle would appear; an unpredictable manner was utilized to present the blocks of visual cues. When the participant made a mistake, the stimulus would remain at the same position until the participant chose the correct answer (Lévesque, Théoret, & Champoux, 2014). The order of 12 visual cues in each trial was never the same in two subsequent stimuli. An effort was made to equalize the ratio of digit presses across 12-stimulus trial (3:3:3:3) (Nitsche et al., 2003).

In the first day of testing, two blocks of random and predetermined sequence were used to familiarize the participants with the task, which was followed by 8 training blocks. Also, the 8 training blocks were carried out on days 2 and 3 of the training. Four weeks after the intervention, the retention test included two sequential blocks was administered in a follow-up assessment session to measure the durability of a-tDCS effects (Figure 2).

### 2.4. Transcranial direct current stimulation

TDCS was applied through two saline-soaked surface sponge electrodes driven by a battery producing direct current (ActivaDose®II. Iontophoresis Delivery Unit, USA). For each experimental condition, study participants received a-tDCS in three consecutive days in a random order while they were blinded to the experimental conditions (active or sham).

**Figure 3.**
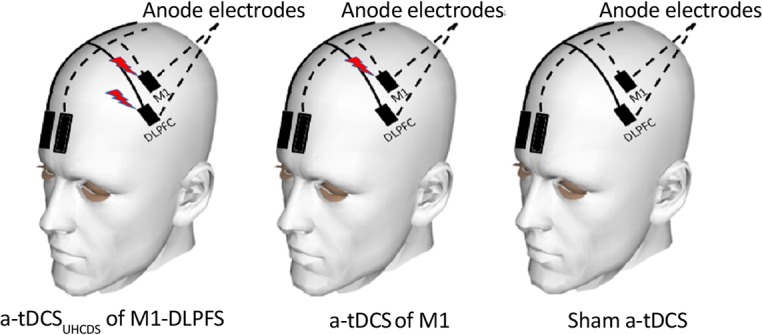
Electrode montages for M1 and UHCDS of M1-DLPFC a-TDCS The active electrodes were placed over left M1 and DLPFC for a-tDCS of M1-DLPFC, and over left M1 for a-tDCS of M1. The reference electrodes were positioned over the contralateral supraorbital area in all groups. In the sham a-TDCS group, the same montages were used as for M1-DLPFC or M1 tDCS.

Based on the international 10–20 system, two active anode electrodes (2×3 cm) were located over left DLPFC and M1 for the experimental (a-tDCSUHCDS), control (M1 atDCS), and sham groups. Two return electrodes (2×6 cm) were also placed over the contralateral supraorbital area (Vaseghi et al., 2015a) (Figure 3). Therefore, two single-channel stimulator instruments were employed in all experimental conditions. Two instruments joined to the active electrodes over M1 and DLPFC were switched on in the experimental group, while only the instrument joined to the active electrode over M1 was switched on in the control group, and one or two instruments were pseudo-randomly switched on for only 60 seconds in the sham group.

The small size of active electrodes enabled us to have more focused effects (Bikson, Datta, Rahman, & Scaturro, 2010; Nitsche et al., 2007; Vaseghi et al., 2015a). On the other hand, the size of return electrode was kept larger to decrease the density and therefore induce less effect under these electrodes (Bastani & Jaberzadeh, 2013; Nitsche et al., 2007). In order to minimize the side effects, the tDCS stimulators were arranged to supply 1 mA DC for 20 minutes, with 10 seconds of ramp-up and ramp-down (Brunoni et al., 2011; Nitsche & Paulus, 2000).

### 2.5. Evaluation of the side effects

To evaluate unpleasant effects, all participants were requested to report the side effects of stimulation such as itching, tingling, burning, headache, discomfort, or pain sensation by Numeric Analog Scales (NAS) during and after tDCS intervention (e.g. 0 is estimated as minimal tingling and 10 as the maximal tingling) (Boros, Poreisz, Münchau, Paulus, & Nitsche, 2008; George & Aston-Jones, 2010).

### 2.6. Operational definitions

The Response Time (RT) is defined as the mean time taken by participants from the appearance of the stimulus on the screen to press the correct key. It was measured for 12 stimuli within each trial and overall for 10 trials within each block. RT of less than 200 ms or more than 3000 ms or those exceeding more than three Standard Deviations (SD) above the individuals` mean were discarded (Nitsche et al., 2003). In addition, the error rate was measured for each block. Error rate was interpreted in accordance with the percentage change in the total number of error responses over 10 trials.

There is a trade-off between the speed and accuracy and it is expected that when speed increases, accuracy decreases and vice versa during the SRTT. Improvement in trade-off between speed and accuracy is referred to as skill. Therefore, Skill Index (SI) considers both the speed and accuracy parameters during the task (Cuypers et al., 2013; Saucedo Marquez et al., 2013). Thus, the main outcome measurement for motor learning assessment was changes in the skill. SI for SRTT was calculated by the following formula:

$$SI=\frac{\mathrm{Percentage}\;\mathrm{of}\;\mathrm{correct}\;\mathrm{sequences}}{\mathrm{Mean}\;\mathrm{responses}\;\mathrm{times}\;\mathrm{per}\;\mathrm{each}\;\mathrm{block}}$$

In the current study, any difference in the skill acquisition, which occurred at last block of day 3 (post-intervention time point), was regarded as the behavioral outcome for the evaluation of motor skill acquisition. Moreover, long-term retention was considered as any change in the skill that occurred 4 weeks after cessation of the intervention (retention time point).

### 2.7. Statistical analyses

The data were blindly analyzed by applying SPSS version 22. To evaluate the normal distribution of data, the variables were examined by Kolmogorov-Smirnov (K-S) test. One-way Analysis of Variance (ANOVA) was used to compare any significant difference in baseline values among groups. The effects of two independent variables, i.e. the groups (experimental, control, and sham) and time points (baseline, posttest on day 3, retention time), on motor skill learning were evaluated through two-way repeated measures ANOVA.

Mauchly’s test was carried out to indicate the validity of the sphericity assumption for repeated measures ANOVA. Greenhouse-Geisser corrected significance values were applied when sphericity was lacking (Meyers, Gamst, & Guarino, 2006). Moreover, a paired-sample t test using the Least Significant Difference (LSD) adjustment was applied to test motor skill learning at post-intervention and retention time points in each group. To determine if participants were effectively blinded to the tDCS groups (active or sham), the Pearson’s chi-square test was carried out. Alpha level was set at less than 0.05. In addition, the power of test was considered 0.80. All results are displayed as Means±Standard Error of Measurement (SEM).

## 3. Results

### 3.1. Comparison of baseline values

All variables in all groups were normally distributed according to K-S test. Table 1 shows demographic details and baseline data for the participants in three groups. Based on the results, there were no statistically significant differences among participants in different groups regarding the gender, age, MMSE, and baseline skill scores (P>0.05) (Table 1).

**Table 1.** Demographic data and baseline values of the participants (Mean±SEM)

**Variable**	**Group (Mean±SEM)**			**Sig.**

	**Experimental (n=20)**	**Control (n=16)**	**Sham (n=17)**	
Gender (male, female)	6,14	3,13	4,13	0.73
Age, y	27.90±0.88	28.19 ±1.00	27.70±0.88	0.94
MMSE test	29.70±0.13	29.56 ±0.18	29.59±0.19	0.82
Skill	0.19±0.01	0.18±0.008	0.18±0.008	0.47

### 3.2. Changes in behavioral outcomes over multiple sessions of motor training

**Figure 4.**
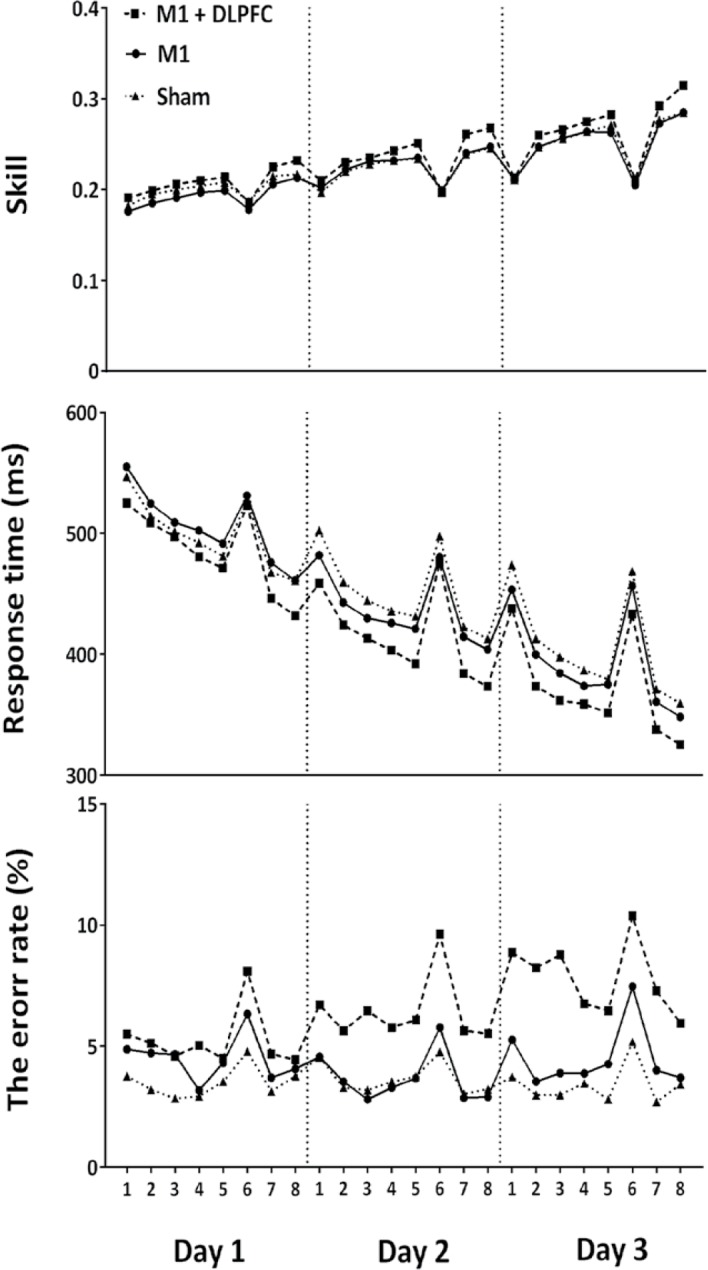
The average RT, error rate and SI for each block in all groups Skill measure, response time and error rate (Mean±SEM) for M1+DLPFC (black squares), M1 (black circles) and sham (black triangles) a-tDCS groups are shown in 8 training blocks for day 1, day 2, and day 3 of the training.

The average RT, error rate and SI for each block in all groups are presented in Figure 4. As it can be seen in this figure, all groups exhibited a trend toward increase in SI and decrease in RT for 8 training blocks over three days of practice. This coincided with a larger error rate in a-tDCSUHCDS group as compared to M1 or sham tDCS group (Figure 4).

### 3.3. The results of a-tDCS on skill learning

Table 2 presents the outputs of a mixed-model repeated measures ANOVA. The interaction effect between time and group illustrates differences in the trend of learning among stimulation groups at some time points that are statistically significant (P<0.05) (Table 2).

**Table 2.** ANOVA results for the effects of a-tDCSUHCDS on the skill

**Outcome Measure**	**Main and Interaction Effects of ANOVA**	**df**	**F**	**Sig.**
Skill	Time (within-subject effects)	1.46	72.93	<0.001^*^
	Time^*^ group (interaction effects)	2.92	2.87	0.04^*^
	Group (between-subject effects)	2	2.33	0.11

* Indicates significant difference at 0.05 level.

**Figure 5.**
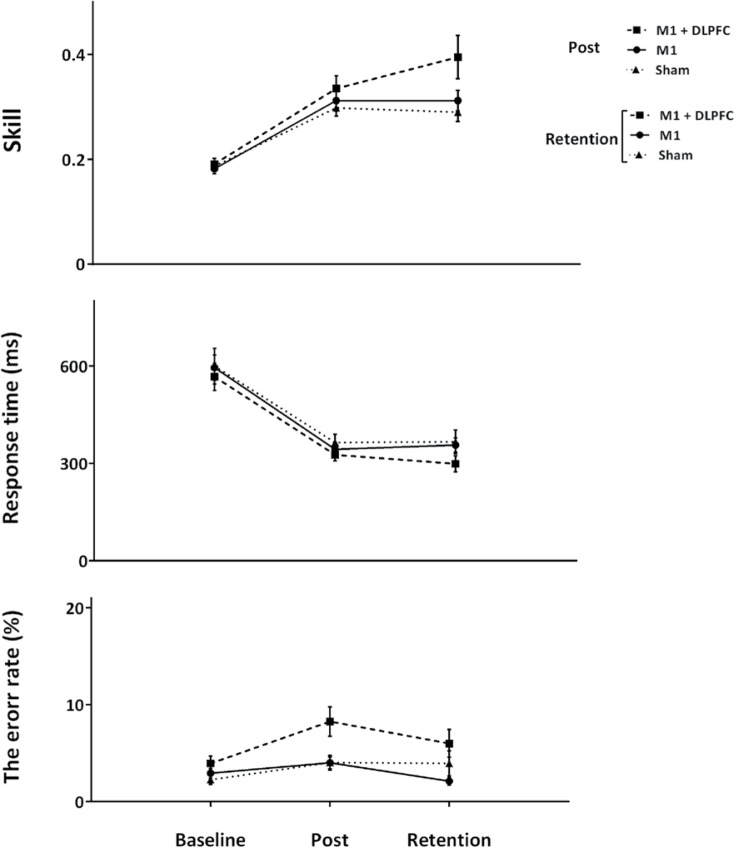
The average RT, error rate and SI for time points in all groups Retention of skill, retention of response time and retention of error rate (Mean±SEM) for M1+DLPFC (black squares), M1 (black circles) and sham (black triangles) tDCS groups are shown at first block of day 1 (baseline), last block of day 3 (post-intervention) and 4 weeks after cessation of intervention (retention). The bracket shows a significant difference between two groups at retention time point.

Multiple comparisons using the LSD adjustment revealed no significant differences between a-tDCSUHCDS and control or sham group at post-intervention (P>0.05) (Figure 5). In accordance with the findings, a significant difference was seen between a-tDCSUHCDS and sham tDCS at retention time (P<0.05) (Figure 5), while there were no significant differences between M1 and both a-tDCSUHCDS and sham tDCS conditions at retention time (P>0.05) (Figure 5). As Figure 5 demonstrates, the positive effect of a-tDCSUHCDS lasted for 4 weeks after cessation of the intervention.

A two-tailed, paired sample t test with LSD adjustment indicated a significant difference between retention assessment and post-intervention in a-tDCSUHCDS and M1 atDCS conditions (P<0.05), while there was no significant difference between retention assessment and post-intervention in sham tDCS (P>0.05) (Table 3). Moreover, there were significant differences among all three groups regarding the post-intervention and baseline values (P<0.001) (Table 3).

**Table 3.** Pair-wise comparison of skill with LSD adjustment between the time points in each group

**Group**	**Variable**	**Time**	**Time**	**Mean Differences (95% CI)**	**Sig.**
A-tDCSUHCDS	Skill	Baseline	Post-intervention	−0.12 (−0.16, −0.08)	<0.001^*^
		Post-intervention	Retention	−0.08 (−0.15, −0.01)	0.02^*^
M1 a-tDCS	Skill	Baseline	Post-intervention	−0.11 (−0.13, −0.09)	<0.001^*^
		Post-intervention	Retention	−0.03 (−0.05, −0.008)	0.01^*^
Sham a-tDCS	Skill	Baseline	Post-intervention	−0.10 (−0.13, −0.08)	<0.001^*^
		Post-intervention	Retention	−0.005 (−0.02, 0.01)	0.60

* Indicates significant difference at 0.05 level.

### 3.4. Side effects of a-tDCS

All three a-tDCS groups rated their sensations under active (anode) electrode and reference (cathode) electrode through three stages of stimulation, including start (0–7 min of stimulation), middle (7–14 min of stimulation) and completion (14–20 min of stimulation) on day 1, day 2 and day 3, respectively (Tables 4 and 5).

**Table 4.** Numeric sensation scores during experimental conditions under anode electrode

**Sensation**		**M1 tDCS**			**UHCDS tDCS**			**Sham**		

		**Day 1**	**Day 2**	**Day 3**	**Day 1**	**Day 2**	**Day 3**	**Day 1**	**Day 2**	**Day 3**
Tingling	Beginning	4.33±0.18	4.33±0.20	4.27±0.21	4.95±0.14	4.90±0.20	4.75±0.21	2.44±0.12	2.38±0.11	2.22±0.10
	Middle	2.83±0.16	2.88±0.17	3.16±0.21	3.21±0.12	3.07±0.12	3.35±0.18	1.22±0.10	1.02±0.09	1.05±0.10
	End	1.11±0.18	1.28±0.21	1.33±0.20	1.47±0.11	1.50±0.11	1.50±0.13	0.89±0.18	0.89±0.20	1±0.25
Itching	Beginning	2.83±0.35	2.88±0.36	3± 0.37	2.85±0.33	2.75±0.33	2.75±0.32	1.38±0.29	1.50±0.29	1.61±0.31
	Middle	1.66±0.28	1.77±0.29	2± 0.29	1.90±0.23	1.85±0.29	1.75±0.28	0.66±0.22	0.50±0.18	0.44±0.18
	End	0.83±0.23	0.55±0.18	0.61±0.24	0.90±0.23	0.90±0.22	0.80±0.23	0.66 ±0.24	0.33±0.14	0.28±0.18
Burning	Beginning	0.16±0.16	0.55±0.30	0.66±0.31	0.47±0.28	0.85±0.36	0.95±0.38	-	-	-
	Middle	-	-	-	-	-	-	-	-	-
	End	-	-	-	-	-	-	-	-	-

Scores are reported as Mean±SEM (SEM: Standard Error of Measurement).

**Table 5.** Numeric sensation scores during experimental conditions under reference electrode

**Sensation**		**M1 tDCS**			**UHCDS tDCS**			**Sham**		

		**Day 1**	**Day 2**	**Day 3**	**Day 1**	**Day 2**	**Day 3**	**Day 1**	**Day 2**	**Day 3**
Tingling	Beginning	2.05±0.10	2.16±0.16	2.05±0.18	2.02±0.11	2.20±0.18	2.20±0.16	1.83±0.14	2±0.20	2±0.19
	Middle	1.55±0.08	1.55±0.09	1.61±0.11	1.78±0.05	1.60±0.07	1.67±0.09	1.72±0.07	1.63±0.07	1.61±0.10
	End	0.66±0.08	0.80±0.10	0.80±0.11	0.83±0.05	0.80±0.05	0.77±0.07	0.72±0.07	0.80±0.06	0.75±0.08
Itching	Beginning	2± 0.30	2.11±0.30	2.11±0.29	1.95±0.24	1.95±0.25	2±0.25	1.77±0.20	1.83±0.21	1.72±0.21
	Middle	1.11±0.17	1.27±0.22	1.33±0.22	1.19±0.17	1.25±0.21	1.20±0.21	1.16±0.18	1.27±0.24	1.16±0.21
	End	0.27±0.10	0.27±0.11	0.50±0.20	0.47±0.13	0.55±0.17	0.40±0.17	0.39±0.14	0.50±0.18	0.39±0.18
Burning	Beginning	-	-	-	-	-	-	-	-	-
	Middle	-	-	-	-	-	-	-	-	-
	End	-	-	-	-	-	-	-	-	-

Scores are reported as Mean±SEM (SEM: Standard Error of Measurement).

According to the participants’ reports, itching and tingling were the most important side effects under both active and reference electrodes. Three of the participants in the a-tDCSUHCDS group and two of the participants in the M1 a-tDCS group reported burning sensation at the beginning of the stimulation. There were no other side effects such as discomfort or pain through three stages of brain stimulation over three consecutive days. In addition, the perceptions of participants on the stimulation condition after the last stimulation session are summarized in Table 6. The results of Pearson’s chi-square test revealed no significant differences between the active and sham tDCS groups (χ^2^=3.73, P=0.44). The active or sham tDCS groups properly guessed only in 13.2% of groups (except the “cannot say” responses) (Table 6).

**Table 6.** The participant’s perception on the stimulation condition after the last stimulation session

**Perception of Participants**		**M1 a-tDCS**	**UHCDS a-tDCS**	**Sham a-tDCS**	**Total**
Perceived stimulation	Active	2	3	5	10
	Sham	5	7	2	14
	Cannot say	9	10	10	29
	Total	16	20	17	53

## 4. Discussion

The results of the present study indicate a significant difference between the a-tDCSUHCDS and sham tDCS groups in long-term retention (4 weeks after the intervention). The results also indicate that all individuals experienced an improvement in the skill and RT after the training course. Thus, multiple sessions of a-tDCS during the training led to a durable effect up to 4 weeks; however, a-tDCSUHCDS had a longer lasting (up to 4 weeks) effect on learning.

Probably due to cumulative effects, multiple sessions of a-tDCS create greater and longer lasting behavioral effects (Gershon, Dannon, & Grunhaus, 2003). According to the results of the present study, after completion of the last training session, no significant difference regarding skill was observed between a-tDCSUHCDS and control or sham groups. These results are in agreement with the previous studies which reported that immediately after the last training session there was no significant difference between motor learning following application in the M1 a-tDCS and sham tDCS groups (Boggio et al., 2006; Cuypers et al., 2013; Ehsani et al., 2016; Galea et al., 2011; Zimerman et al., 2013). On the contrary, it was reported that M1 a-tDCS causes a significant improvement in motor learning as compared to sham tDCS (Nitsche et al., 2003).

This difference may be attributed to the methodological differences between that study and the present one. As opposed to the present study in which small electrodes (6 cm^2^) were utilized, larger electrodes (35 cm^2^) were used in the study carried out by Nitsche et al. (2003); therefore, M1 stimulation may also be contaminated with the stimulation of nearby cortical sites. The multiple session nature of the present study was another difference between the two studies.

The findings in the current study indicated that a-tDCSUHCDS induced greater motor skill learning as compared to sham tDCS over the follow-up period of 4 weeks, while this considerable long-lasting effect was not spotted between the M1 and sham tDCS groups at 4-week retention. It appears that offline changes after the last intervention session are a critical factor for the length of lasting effects in atDCS studies. Some a-tDCS studies assessed the results up to 24 hours after the stimulation completion (Kang & Paik, 2011; Kantak et al., 2012), since the present study assessed the lasting of the results for up to 4 weeks.

To the best of our knowledge, Reis et al. and Marquez et al. studies are the only multiple session studies that reported prolonged enhancement of effects of simultaneous conventional M1 a-tDCS and motor training in healthy individuals (Reis et al., 2009; Saucedo Marquez et al., 2013). In contrast, the current study indicated long-lasting (up to 4 weeks) results of multiple sessions of a-tDCSUHCDS of M1-DLPFC on skill acquisition. Literature reveals that increased DLPFC activity is correlated with increased M1 activity (Vaseghi, Zoghi, & Jaberzadeh, 2015b; Wager et al., 2004). Vaseghi et al. (2015b) reported that a-tDCS of DLPFC significantly increased M1 CSE. This indicates functional connectivity between these cortical sites (Baudewig, Nitsche, Paulus, & Frahm, 2001; Lang, Nitsche, Paulus, Rothwell, & Lemon, 2004; Lang et al., 2005).

Literature review suggests a functional relationship between DLPFC and M1 (Hoshi, 2006; Kolb & Whishaw, 2009). It may have a key role in long-term retention of the learning task. It can be stated that using multiple sessions of simultaneous stimulation of such functionally-connected cortical sites leads to a considerable increase in the neurons firing rate and the chance of recently-established connections between activated neurons during SRTT that can influence learning consolidation and retention for 1 month (De Xivry & Shadmehr, 2014; Nitsche et al., 2003).

Alternatively, it could be argued that the different lasting effects between previous studies (Kang & Paik, 2011; Kantak et al., 2012; Reis et al., 2009; Saucedo Marquez et al., 2013) and our study was due to different learning tasks that comprised either of single session or multiple sessions of practice, retest interval time after the end of training, as well as other methodological differences regarding a-tDCS parameters.

We also hypothesized that the amount and lasting effects of a-tDCSUHCDS would be more than conventional a-tDCS of M1 on motor learning. The findings in the current study could not support this hypothesis. The findings indicated no significant differences in overall skill acquisition between a-tDCSUHCDS and M1 a-tDCS. In the current study, the effect size of a-tDCS was 0.53 between a-tDCSUHCDS and M1 a-tDCS groups at retention time that indicates a moderate effect. In the present study, increase in sample size might lead to different findings with regard to the retention time between a-tDCSUHCDS and conventional M1 a-tDCS.

Another important finding of this study is that acquisition and consolidation of motor learning are different from those of several other studies that reported reduction of the error rate following application of M1 a-tDCS (Vines, Cerruti, & Schlaug, 2008; Vines, Nair, & Schlaug, 2008; Zimerman et al., 2013). We found that although a-tDCSUHCDS induced a significant improvement in skill, there were considerable increase in error rate in a-tDCSUHCDS as compared to both M1 and sham tDCS. The trade-off between speed and accuracy affects most learning tasks such as SRTT. It seems that due to the increased speed in a-tDCSUHCDS, the participants should “pay the price” by increase in error rate during SRTT. Moreover, these might be due to attention level differences between individuals.

The findings of this study also indicate that the application of a-tDCSUHCDS and also M1 a-tDCS with the small active electrode sizes (6 cm^2^) is safe and only induces minimal side effects. In line with previous studies (Brunoni et al., 2011; Gandiga, Hummel, & Cohen, 2006), itching and tingling were the most outstanding side effects. Except the mentioned side effects, there were no other unpleasant effects such as headache, pain or nausea during or after brain stimulation. It should be noted that 5 out of 53 participants reported a burning sensation.

We acknowledge that there are some limitations in the present study. The sample size in the present study was small. Therefore, it is recommended that larger samples of healthy individuals be considered in the future studies to support these findings. Moreover, most individuals in the present study were women; therefore, it is difficult to generalize the conclusions to men. It is also important to carry out investigations into gender differences with larger sample size in responses to a-tDCSUHCDS. In addition, generalizing the findings to older individuals or patients with neurological disorders is impossible. Thus, it is recommended that more research evaluate the effects of multiple sessions of a-tDCSUHCDS on the extent and durability of the motor sequence acquisition in older population or patients. Accordingly, further understanding of optimal tDCS parameters and the mechanisms behind the effects should be pursued to achieve better clinical outcomes.

## Ethical Considerations

### Compliance with ethical guidelines

All ethical principles were considered in this article. The participants were informed about the purpose of the research and its implementation stages; They were also assured about the confidentiality of their information; Moreover, They were allowed to leave the study whenever they wish, and if desired, the results of the research would be available to them. (Ethical code: IR.USWR.REC.1394.222)
